# Imageless navigation system (Naviswiss) provides accurate component position in total hip arthroplasty with lateral decubitus position for end-stage hip osteoarthritis: a prospective cohort study with CT-validation

**DOI:** 10.1186/s42836-023-00224-0

**Published:** 2024-01-08

**Authors:** Corey J. Scholes, Manaal Fatima, Tobias Schwagli, David Liu

**Affiliations:** 1 EBM Analytics, Sydney, NSW 2065 Australia; 2 Medivation, 5200 Brugg, Switzerland; 3 Gold Coast Centre for Bone and Joint Surgery, Palm Beach, QLD 4221 Australia

**Keywords:** Hip arthroplasty, Imageless navigation, Lateral decubitus, Validation

## Abstract

**Aims:**

The Naviswiss system (Naviswiss AG, Brugg, Switzerland) is a handheld imageless navigation device used to improve the accuracy of implant positioning in total hip arthroplasty (THA). However, clinical data for leg length discrepancy and femoral offset is lacking, and the validity of the system has not been reported for patients undergoing THA in the lateral decubitus position. This study aimed to report the accuracy of the device in this patient population.

**Methods:**

Patients underwent THA in the lateral decubitus position performed by a single surgeon. Component position measured by the device intraoperatively was compared to postoperative measurements on computed tomography (CT) scans. Agreement between the navigation system and postoperative measurements was reported for acetabular cup inclination, acetabular cup version, femoral offset, and leg length discrepancy.

**Results:**

Thirty-three patients were included in the analysis. The mean difference between intraoperative and postoperative CT measurements was within 2° for angular measurements and 2 mm for leg length. Absolute differences in the two indices were up to 4° and 3 mm. The mean bias was 1°–2° overestimation for cup orientation and up to 2 mm overestimation for leg length change. However, 95% limits of agreement did not exceed absolute thresholds of 10° and 10 mm, especially after correction for bias. One case (3%) was declared intraoperatively for issues with fixation on the greater trochanter.

**Conclusions:**

The accuracy of the Naviswiss system falls within clinically acceptable recommendations for acetabular cup placement, femoral offset, and leg length for total hip arthroplasty with a anterolateral approach in lateral decubitus position. The system could be further improved with regression-based bias correction.

**Supplementary Information:**

The online version contains supplementary material available at 10.1186/s42836-023-00224-0.

## Introduction

Accurate component placement is an important factor impacting outcomes of total hip arthroplasty (THA) [4]. Inappropriate positioning of the femoral stem or acetabular cup is associated with impingement, instability, bearing failure, leg length discrepancy, and an increased risk of the need for revision surgery [5, 18, 24].

Efforts to guide component placement in THA include robotic-assisted surgery and computer-navigated systems [3]. Computed tomography (CT)-based navigation relies on preoperative planning on CT images, which increases both cost and radiation exposure, while imageless navigation is based on landmark registration and does not take into account detailed patient-specific anatomy [30]. Studies have shown that imageless navigation could increase the accuracy of acetabular implant positioning [21], but surgical time remains markedly longer [15] with no clinically meaningful differences in patient-reported outcomes [29]. Despite being in use for over 20 years, the expenses associated with the purchasing and maintenance of navigation systems, the usability and longer operation time have been preventing their widespread use [25].

Advances in imageless technology have seen the development of portable navigation systems, including accelerometer-based devices, which have the advantage of improved accuracy of implant positioning [27] and have been tested with various surgical approaches [2, 8, 12]. The Naviswiss system (Naviswiss AG, Brugg, Switzerland) is a handheld imageless navigation device that utilizes an infrared stereo camera and an inertial measurement unit to calculate the position and orientation of the pelvis, greater trochanter and camera in space [6]. It has been tested in vivo with an anterolateral approach in the supine position, with a < 3° mean absolute error for cup inclination and anteversion [10]. The system has also been tested using a direct anterior surgical approach with fluoroscopy, and the absolute difference between intraoperative and radiographic measures was < 3.5° for cup orientation [23]. However, clinical data for leg length discrepancy and femoral offset is lacking, and the accuracy of the system has yet to be reported for patients in the lateral decubitus position. Hip navigation systems have, in the past, been difficult to use with hip approaches in the lateral decubitus posture due to issues with registration of the anterior pelvic plane.

A trial protocol was developed to assess the validity of this new and unique hip navigation system in measuring acetabular cup inclination, acetabular cup version, femoral offset and leg length discrepancy [6]. This study reports the accuracy of the device by comparing intraoperative component position to postoperative CT measurements, specifically in patients undergoing THA using an anterolateral approach in the lateral decubitus position. We hypothesized that the handheld navigation system would provide precise intraoperative information for acetabular cup orientation, as well as leg length and offset change following primary THA in the lateral decubitus position.

## Methods

### Patient selection

Ethical approval for the study was obtained from the Ramsay Health Care Queensland Human Research Ethics Committee (HREC Reference 20/08), and the study was registered with the Australian New Zealand Clinical Trials Registry (ACTRN12620000873921). Patients over 18 years of age were invited to participate in the study if they presented to the participating surgeon with end-stage osteoarthritis or inflammatory arthritis and underwent THA surgery via an anterolateral surgical approach in the lateral decubitus position. A sample size of 34 cases was established to provide adequate power to detect a 2.5° absolute mean difference between intraoperative navigation results and postoperative CT measurements in the trial protocol [6].

Patients were excluded from the study if they (1) were unable to provide informed consent; (2) had declined or revoked consent for use of clinical data for the study; (3) suffered from severe contralateral hip deformity or dysplasia; (4) required a simultaneous bilateral procedure; (5) required an ipsilateral revision procedure; (6) had a short-stem component implanted; or (7) were lost to follow-up.

### Surgical technique and intraoperative measurement of component positioning

All patients underwent THA by a single surgeon using identical surgical techniques and perioperative protocol. Preoperative templating was performed using a 3D CT assessment and an automated 3D planner (Formus Labs, Auckland, New Zealand). The 3D planner predicted the femoral component according to endosteal fit within the femur and aimed for combined anteversion of 37° by adjusting the cup version.

Patients either received a spinal anaesthesia with sedation or general anaesthesia, or general anaesthesia alone with muscle relaxant under the discretion of the anaesthetist. The patients were positioned in the lateral decubitus position and held stable with standard hip bolster supports at the sacrum and anterior superior iliac spines. The pelvic position was checked by the operating surgeon to ensure a stable vertically-parallel anterior pelvic plane prior to draping. Two 3-mm pins were inserted into the iliac crest to secure the pelvic tracker and a mini-plate secured with a single 3.5-mm locking screw of 25 mm in length was used to attach the femoral tracker to the greater trochanter of the femur. The femoral tracker could be removed and reattached to the locking plate when needed for leg length and offset measurement. The Naviswiss camera was draped and secured to a small trolley on the contralateral side of the surgeon. The camera was operated by a surgical assistant.

The hip was exposed through an anterolateral approach, and the anterior 50% of the abductor insertion was detached just anterior to the musculotendinous junction, keeping the gluteus medius and minimus as one flap. All landmarks were registered according to the standard workflow, including the hip centre of rotation (COR), anterior and posterior attachments of the transverse acetabular ligament and superior acetabular rim. The COR was identified by the functional method [16], with the thigh moved through a multiplanar range of motion while the tags were tracked with the handheld camera. The hip was dislocated anteriorly and following bone preparation, uncemented acetabular and femoral components were inserted. All patients received an E1 Poly liner and a ceramic head. The cup position was registered after stable impaction on the acetabular shell. The final length leg and offset were measured after the hip was relocated with the real femoral head. The final intraoperative component positions were logged by the navigation system and exported for analysis of acetabular cup inclination (ACI), acetabular cup version (ACV), femoral offset (FO), and leg length discrepancy (LLD). All surgeries were performed by the senior author.

### Measurement of component position

The primary study outcomes were extracted for analysis as previously described [6]. Agreement between the navigation system applied intraoperatively and postoperative measurements were assessed using the following parameters:

- ACI: The angle between the acetabular and longitudinal axes when projected onto the functional pelvic plane (FPP);
- ACV: The angle between the acetabular axis and the FPP;
- FO: The relative difference between the hip centre of rotation of the operated joint relative to its starting position at the initial assessment on the coronal plane (medial–lateral) within the pelvic coordinate system;
- LLD: The change in the distance between the greater trochanter tag and the hip centre of rotation summed with the change in the distance between the centre of the acetabulum and the centre of the cup on the transverse plane (superior-inferior)

Blinded images in DICOM (Digital Imaging and Communications in Medicine) format were used for all pre- and postoperative CT measurements of component positioning, with information related to the specific diagnosis, study, surgeon or whether navigation used for the hip arthroplasty procedure removed prior to the measurement of component position. The pre- and postoperative images were blinded by an independent research assistant who was not involved in performance of the measurements.

The postoperative component position was measured by loading the DICOM data to dedicated software (3D Slicer, www.slicer.org) to measure the version and inclination of the acetabular cup. FO and LLD were measured through assessment of anatomical landmarks picked in pre- and postoperative scans. For the postoperative CT assessments, coordinate systems for the pelvis and femur were determined based on the anatomic landmarks. Parameters from both the Naviswiss and CT analysis system were expressed relative to the FPP, with the origin placed at the centre of the line connecting the left and right anterior superior iliac spine (ASIS). For the postoperative CT analysis, the position of the cup centre was compared with the native hip COR determined from the preoperative CT. FO and LLD were reported as the pre-to-post change of the femoral coordinate frame relative to pelvis FPP coordinates on the coronal (mediolateral) and transverse (inferior-superior) planes respectively.

## Data and statistical analysis

### Missing data

Height and weight were unavailable for one case and the mean body mass index (BMI) of the group was used to address the missing value to enable regression on the full dataset.

### Summary agreement

The mean deviation (delta) was calculated by subtracting the imaging measurement from the intraoperative measurement. A positive result indicated an overestimation by the navigation system, and a negative value denoted an underestimation. Bootstrap analysis for delta was performed for each measurement (ACI, ACV, FO, LLD) with 1,000 replications with replacement and a fixed initial seed. Mean, standard error, standard deviation, 95% confidence intervals for the mean were calculated. A Bland–Altman plot was generated for each measure using the limits of agreement (LOA) calculated with the following formula [1, 7]:

$$\mathrm{LOA }=\mathrm{ mean}\pm (\mathrm{standard\ deviation }\times 1.96)$$

Linear regression was used to assess the relationship between mean deviation (delta) and the average of the intraoperative and imaging measurements.

Delta was converted to absolute value and the summary statistics were calculated with the bootstrap analysis as described.

### Bias assessment and correction

Linear regression with bootstrapping was used to assess the relationship between delta and the intraoperative measurement, adjusted for age at surgery, BMI and sex. Model predictions based on the original intraoperative measurements were used to create a bias-adjusted version of the intraoperative measurements and a bias-corrected delta calculated by subtracting the imaging measurements from the bias-corrected intraoperative measurements. Alternative correction for offset and leg length change was performed by dropping values where an intraoperative declaration was made and the summary agreement analysis repeated as described above (Supplementary material 1). All statistical analyses were performed using Stata (v17.1, Statacorp, College Station, TX, USA), with alpha set at 5% to indicate significant effects where appropriate.

## Results

### Patient characteristics

**Fig. 1:**
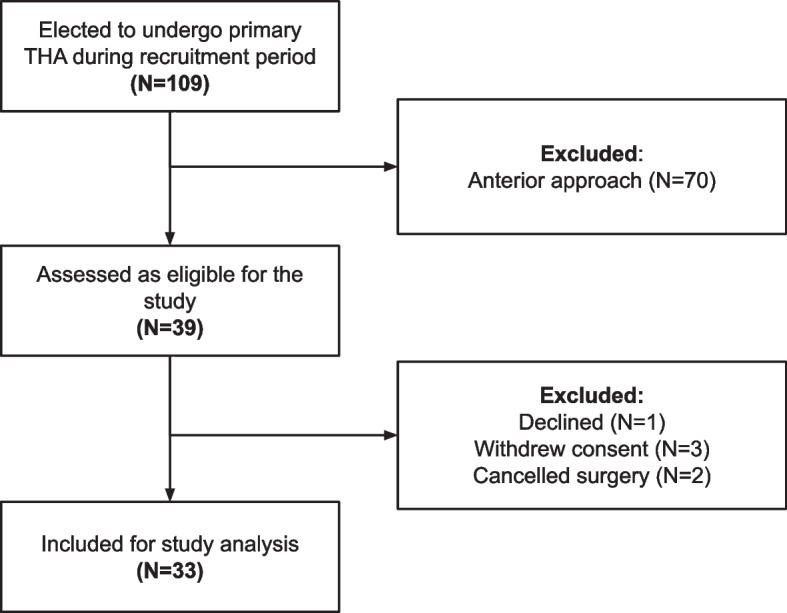
STROBE (Strengthening the Reporting of Observational studies in Epidemiology), diagram [32] of patient inclusion into the study analysis

A cohort of 109 consecutive cases undergoing primary THA were assessed for eligibility, with 33 included for study analysis (Fig. 1). The analysis cohort comprised 52% females and had a mean age at surgery of 68.9 years (SD 8.8) and a mean BMI of 28.2 (SD 4.7).

### Complications

Complications were observed in four cases: one case of periprosthetic infection that underwent washout with liner and head exchange at 8 weeks; one case of stem subsidence that stabilised after 5 months and did not require further surgery and was symptom-free; one case of superficial wound infection that resolved with a course of oral antibiotics and one case of numbness in the contralateral thigh that resolved without intervention.

### Agreement between intraoperative and image-based measurements

#### Thresholds and declared observations

Intraoperative declarations were made for six patients, with reasons of loss of fixation of the tracker on the greater trochanter (*n* = 4) and acquisition failure (*n* = 2) being cited. One patient without an intraoperative declaration exceeded the specified measurement thresholds for both inclination and version, but not for offset or LLD (Table 1).

**Table 1:** Patients without an intraoperative declaration exceeding the measurement thresholds

Measurement	Threshold	Units	Patients above threshold (n)	Proportion above threshold (%)
Threshold-Inclination	10	degrees	1	3
Threshold-Version	10	degrees	1	3
Threshold-Offset	10	mm	0	0
Threshold-LLD	10	mm	0	0

**Fig. 2:**
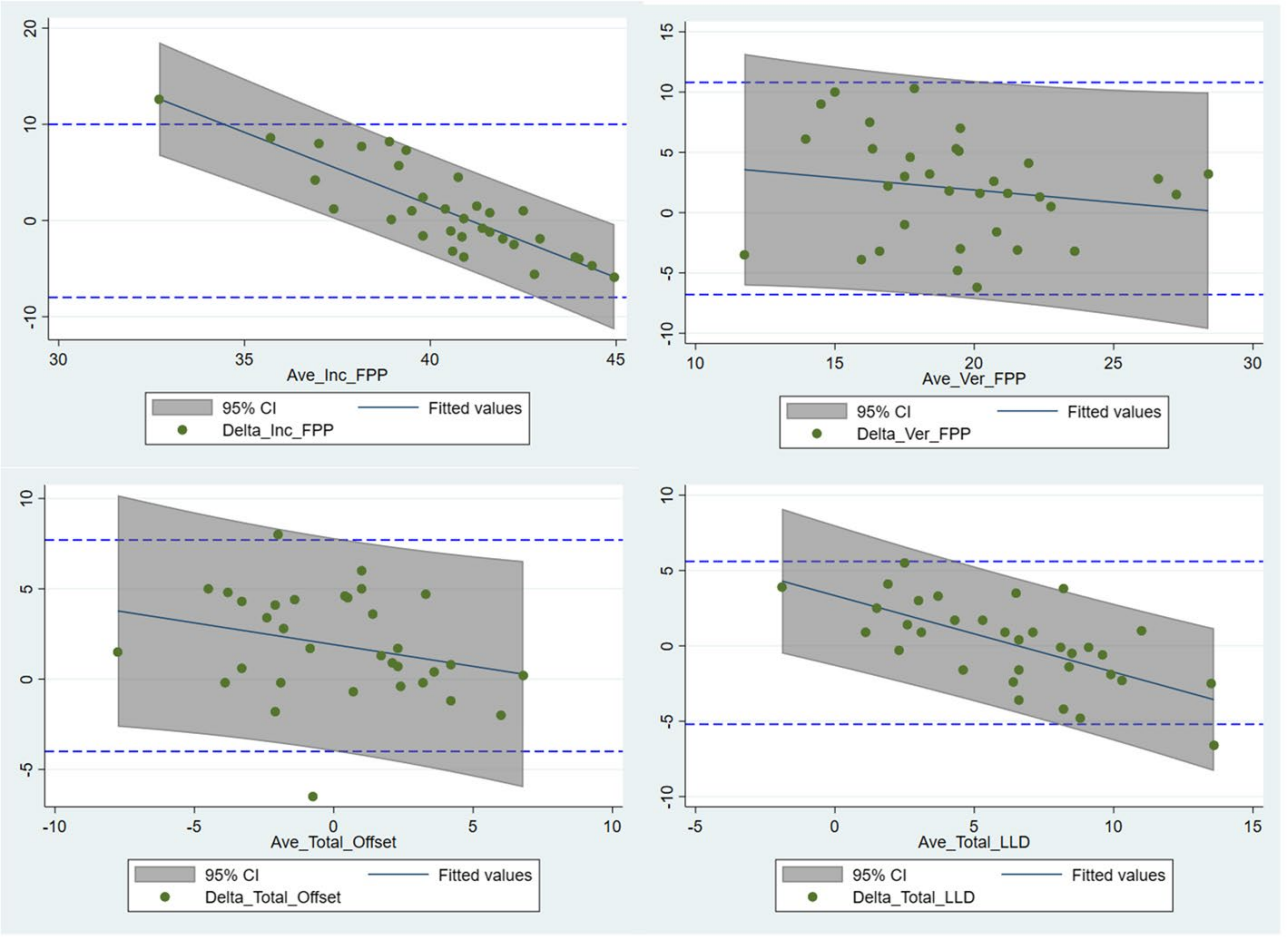
Bland–Altman plots with 95% limits of agreement for inclination and version, offset and leg length (LLD). Regression fits with shaded areas denoting 95% prediction intervals indicate the relationship between magnitude and agreement

**Fig. 3:**
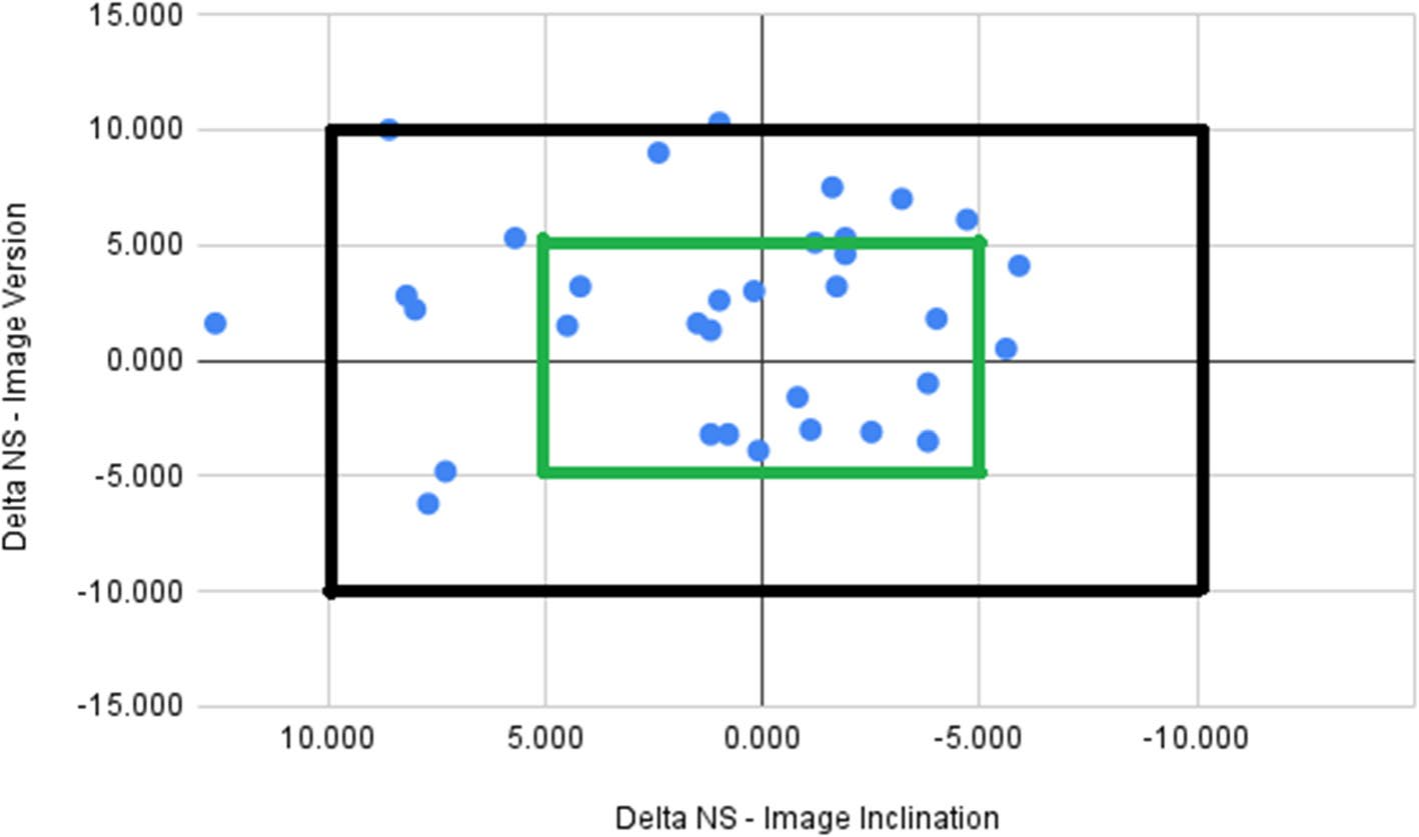
Scatterplot of delta in version versus inclination for all cases. Outer box: 10° threshold; Inner box: 5° threshold

The mean differences between the intraoperative measurements and the postoperative imaging analysis were less than 1° for inclination, less than 2° for version, and less than 2 mm for both offset and LLD (Table 2). The 95% LOA were within 10° for inclination and version, and within 8 mm and 6 mm for offset and LLD respectively (Table 2, Fig. 2). Overall, 90% of cases (95%CI 75.3–98.1) were within 10° of the image-measured result for both inclination and version (Fig. 3). The linear fit of the average to the delta indicated that the bias between the navigation and the CT measurements was not constant across the magnitude for inclination and LLD (*P* < 0.001, Table 3).

**Table 2:** Summary of mean differences between intraoperative and image-based measurements. *P*-value indicates the probability of observing the mean delta (relative to zero) as extreme assuming the null hypothesis is true

Sign	Mean Delta	SE	SD	95% LCI	95% UCI	*P*-Value	Lower LOA	Upper LOA
Inclination	1.0	0.79	4.6	-0.6	2.5	0.528	-8.0	10.0
Version	2.0	0.77	4.5	0.5	3.5	0.027	-6.8	10.8
Offset	1.9	0.51	3.0	0.9	2.9	<0.001	-4.0	7.7
LLD	0.2	0.47	2.7	-0.8	1.1	0.68	-5.2	5.5
**Absolute**								
Inclination	3.6	0.53	3.1	2.6	4.7			
Version	4	0.44	2.6	3.2	4.9			
Offset	2.7	0.38	2.2	1.9	3.4			
LLD	2.2	0.28	1.6	1.7	2.8			

*LLD* leg length discrepancy, *SE* standard error of the estimate, *SD* standard deviation, *LCI* lower confidence interval, *UCI* upper confidence interval, *LOA* limits of agreement

**Table 3:** Linear fit of average of measurements to delta of measurements

	**Coefficient**	**SE**	**95% LCI**	**95% UCI**	***P*-Value**	**Adjusted R**^**2**^
Inclination	-1.51	0.1	-1.70	-1.31	<0.001	0.70
Version	-0.2	0.23	-0.65	0.24	0.368	0.00
Offset	-0.24	0.15	-0.53	0.05	0.102	0.04
LLD	-0.51	0.09	-0.69	-0.32	<0.001	0.41

### Factors associated with agreement and bias correction

BMI, age and sex were tested as the main effects against all measurements of interest. The regression results indicated a significant association (*P* = 0.049) of sex with inclination delta, a magnitude-dependent bias for version (*P* = 0.005) and offset delta (*P* = 0.011) (Supplementary material 2). Bias correction applied to the intraoperative measures removed overall bias and shrank the between-case variation (SD) of delta by 6%–16%, which increased to 7%–42% for absolute values (Table 4). Bias correction also shrank the mean absolute delta by 5%–26% relative to the uncorrected values. Alternatively, by omitting declared observations for offset and leg length (Table 5), mean absolute error was reduced by 18%–33% and between-case variability by 18%–39%.

**Table 4:** Summary of mean differences between intraoperative and image-based measurements for bias corrected intraoperative measures

Sign	Mean Delta	SE	SD	95% LCI	95% UCI	Lower LOA	Upper LOA
Inclination	0	0.69	4.0	-1.4	1.4	-7.9	7.9
Version	0	0.69	4.0	-1.4	1.4	-7.9	7.9
Offset	0	0.43	2.5	-0.8	0.8	-4.9	4.9
LLD	0	0.44	2.6	-0.9	0.9	-5.0	5.0
**Absolute**							
Inclination	3.2	0.47	2.7	2.24	4.08		
Version	3.3	0.37	2.2	2.6	4		
Offset	2	0.22	1.3	1.58	2.45		
LLD	2.1	0.26	1.5	1.58	2.59

**Table 5:** Summary of mean differences between intraoperative and image-based measurements with declaration cases omitted (*n* = 27)

Sign	Mean Delta	SE	SD	95% LCI	95% UCI	*P*-Value	Lower LOA	Upper LOA
Offset	2.1	0.42	2.4	1.26	2.91	<0.001	-2.7	6.9
LLD	0.4	0.41	2.4	-0.44	1.15	0.386	-4.3	5.0
**Absolute**								
Offset	2.4	0.36	2.1	1.69	3.1			
LLD	1.8	0.23	1.3	1.3	2.2			

## Discussion

The main finding of our study is that the handheld hip navigation system (Naviswiss) provides information that can assist in accurate acetabular cup orientation and leg length and offset restoration in the lateral decubitus position. The mean difference between intraoperative and postoperative CT measurements was within 2° for angular measurements and 2 mm for the length measurements. The absolute differences for the two indices were within 5° and 4 mm.

The mean absolute deviation of acetabular inclination (3.6, 95%CI 2.7–4.5) between the navigation system and the CT-based analysis was comparable to the deviation reported by Naito et al., in [22] (2.8, 2.3–3.3), with patients in the supine position, but higher than the pooled deviation (2.6, 2.4–2.8) for previous studies in the lateral decubitus position using CT-based, imageless and accelerometery systems (Supplementary material 3: summary of validation findings). In contrast, anteversion mean absolute deviation (4, 3.1–4.9) was greater than that reported by Hasegawa (2.8, 2.3–3.3) but comparable to pooled deviation of previous studies (3.6, 3.4–3.8). Overall, the mean bias was 1°–2° overestimation for cup orientation and up to 2 mm overestimation for leg length change, with 95% LOA on or below 10° for orientation and 5 mm–10 mm for offset/leg length change. Absolute thresholds of 10° and 10 mm were established a-priori in the study protocol [6] and were not exceeded by 95% LOA, especially after correction for bias. Between-patient variation in published guidelines for cup orientation varies between 5° and 12° for inclination and up to 18° for version [9]. In general, less than 10 mm of LLD is considered acceptable after THA [20]*.* In addition, a simulation study [28] reported impingement and loss of motion range with a 4 mm medialization/lateralization of the cup, although this amount of change was not justified in their methods. The LOA in the present study suggests that the entire patient sample would fall within these tolerances, except for offset, where the 95% LOA exceeded 4 mm. In reality, the clinical tolerance for error will not be distributed evenly across the population, with patients at the extremes (smaller or larger) of target orientation and position requiring greater accuracy to prevent more extreme cup positions.

Comparing the present results to the literature should be done with caution, due to the heterogeneity of case-mix, the systems employed for intraoperative measurements, the methods by which gold-standard data was derived (CT vs. radiograph), as well as analytical methods (pelvic coordinate systems, statistical analysis) and reporting standards. Recent meta-analyses have reported substantial differences in patient demographics, surgical technique [21] and outcome heterogeneity [27] in studies comparing navigated THA to conventional instrumentation. In addition, some validation attempts have used the anterior pelvic plane (APP) coordinate system [11, 33], while the FPP was used in the present analysis. Further, due to the proprietary nature of the systems under consideration, the presence or absence of bias correction or other real-time compensatory calculations in the system software is not reported in all studies. Nevertheless, the reasons for the higher deviation reported in the present study compared to other validation reports in the literature may be related to differences in case mix between studies. Foremost are differences in BMI and indications for surgery. Hasegawa et al. [10] reported on accuracy in a cohort of patients with an average BMI of 24.6 ± 4.9 compared to the present study 28.2 ± 4.7. Other sources of error include the validity and reliability of the CT measurements, which had up to 2°–3° of deviation from true [26], despite high relative reliability [10]. In addition, the intraoperative measurements from the system are rounded to the nearest whole number, which could create up to 1° deviation from the image-based measurement.

A biphasic pattern of magnitude-dependent bias [14] was observed for inclination (FPP) and leg length change. The navigation system tended to overestimate with smaller average measurements and underestimate with larger averages (Fig. 2). While bias correction was able to re-centre the sample around zero and reduce between-patient variation, further work is needed to validate regression-based bias correction algorithms to remove the magnitude-dependency (slope) by adjusting intraoperative measurements, potentially based on patient demographics and/or other factors. In addition, further work may be required to improve tracker fixation for offset and leg length with 4 cases (12%) declared intraoperatively for issues with fixation on the greater trochanter. While the inclusion of these cases generated acceptable accuracy overall, their omission improved between-case variability in accuracy and reduced the LOA for both offset and leg length, highlighting a potential avenue for expanding indications. A previous study [10], mentioned the potential vulnerability of the system to pin fixation on the iliac crest.

The magnitude-dependent bias observed for inclination in the present series may be associated with higher BMI compared to previous studies. The soft-tissue distribution over key bony landmarks of the pelvis, with particular reference to the shift in distribution with changes in patient position during measurement and surgical approach may be a key source of error in this context. Primarily, a thicker layer of soft tissue at key landmarks may affect intraoperative measurements and limit the sensitivity of the system to pelvic anatomical variation. A similar study conducting a CT validation of an accelerometer-based navigation system reported a significant correlation between cup orientation delta and BMI [12], in a sample with lower average BMI (23.7 ± 4.4). Soft-tissue distribution is also related to age and sex [13], and may contribute to the systematic overestimation of inclination in females included in the present series.

A limitation of navigation in hip replacement surgery is its dependence on landmark acquisition, which is itself dependent on patient BMI, soft-tissues and surgical setup (including draping). The system used in the current study was reliant on stable accurate pelvic positioning, which can be a challenge for the surgeon to replicate the FPP established on the CT 3D reconstruction by visualizing the bony landmarks on the operating table. The ability to identify landmarks and the assumptions made about the relationship between pelvic orientation and those landmarks are key to aligning the plane on the table to that generated by the image-based analysis. Like many previous navigation systems, the Naviswiss relies on the FPP for version and inclination values. However, the FPP can be difficult to measure in the lateral decubitus position and alignment between the measured plane (detected by the trackers) and the true plane may diverge during the various steps of the THA procedure during retraction and leg positioning for exposure. Others have alluded to the effect of soft tissue thickness over key pelvic landmarks [17, 34], albeit it can be mitigated by surgeon experience [34]. Still others have observed challenges of landmark identification after draping [19], and this, in combination with greater tissue thickness, may have reduced the sensitivity of the system. Soft-tissue distribution is also related to age and sex [13], and may contribute to the systematic overestimation of inclination for females in the present series. Interestingly, this was not the case in the Hasegawa series, in which 90% of the cohort were female. The reasons for this effect in the present study are not clear but correction of this bias in the intraoperative measurements reduced mean absolute deviation.

The present study described CT-based validation of a navigation system used for THA in end-stage osteoarthritis subjects, with horizontal LOA between intraoperative and CT measurements of inclination, anteversion, offset and leg length. However, the results should be placed in the context of the key limitations inherent in the study. The first is that the results indicated magnitude-dependent bias within the tolerance thresholds set a-priori, undermining the efficacy of summary statistics for delta and horizontal LOA, as they rely on an assumption of no association between delta and the average of measurements [1, 14]. This also complicated the task of comparing these summary statistics with the findings of other studies, which have not reported their data in this detail. Future validation should consider reporting in such a way as to compare data that contain biphasic magnitude-dependent bias more accurately, such as with regression-generated central tendency and between-case variability [1]. Secondly, it remains uncertain what impact patients undergoing imaging at different imaging facilities 6 weeks after surgery may have had on the CT-derived data. The effect of multiple scanners on the accuracy of CT-based landmark identification has not been reported for this specific application. However, other CT-derived features have shown considerable variability between scanners [31]. In particular, variation in femoral rotation during image acquisition between the preoperative and postoperative scans may impact the ability to identify the longitudinal femoral axis, which is an important landmark for femoral offset. Therefore, it is plausible that some observer error could be attributed to the variation in scan quality. Future studies should consider quantifying this potential source of variation in clinical assessments.

## Conclusions

The navigation system assessed in a primary THA cohort of patients with end-stage hip osteoarthritis provided acceptable validity within clinical recommendations for cup placement, femoral offset and leg length in the lateral decubitus patient position. This paper demonstrated that correction of magnitude-dependent biases observed for inclination and leg length change could further improve system accuracy in real-world application. The ability to apply accurate navigation systems to pathological anatomy in the context of variable anthropometry remains an ongoing challenge for clinical practice.

### Supplementary Information

**Additional file 1: Supplementary material 1.** Code.

**Additional file 2: Supplementary material 2.** Regression model results summary.

**Additional file 3: Supplementary material 3.** Summary of validation findings.

## Data Availability

Upon reasonable request to the authors, only de-identified data (i.e., data with sensitive and personal information removed), will be provided as per the ANDS Publishing and Sharing Sensitive Data Guide.
