# Supplementary material for: Imageless navigation system (Naviswiss) provides accurate component position in total hip arthroplasty with lateral decubitus position for end-stage hip osteoarthritis: a prospective cohort study with CT-validation
Source: Arthroplasty. 2024 Jan 8;6:3. doi: 10.1186/s42836-023-00224-0 (PMC10773062; doi:10.1186/s42836-023-00224-0)
Supplement: Supplementary file 2 — Additional file 2: Supplementary material 2. Regression model results summary. [file 42836_2023_224_MOESM2_ESM.pdf]

Supplementary material 2 - Regression Models not used

Table 1: Regression summary delta inclination (FPP)

Linear regression			
	Number of obs	=	33
	Replications	=	100
	Wald chi2(4)	=	6.72
	Prob > chi2	=	0.1517
	R-squared	=	0.2092
	Adj R-squared	=	0.0963
	Root MSE	=	4.4274

delta_in~fpp	Observed coefficient	Bootstrap std. err.	z	P>|z|	Normal-based [95% conf.	interval]
ns_inc	.2209985	.5602995	0.39	0.693	-.8771683	1.319165
bmi	-.2109395	.13427	-1.57	0.116	-.4741039	.052225
ageatsurgery	-.1249936	.1127633	-1.11	0.268	-.3460056	.0960185
sexcode						
M	-3.197874	1.621098	-1.97	0.049	-6.375169	-.0205797
_cons	8.272596	27.49402	0.30	0.764	-45.61469	62.15988

Table 2: Marginal Estimates - Delta Inclination and Sex

	Margin	Delta-method std. err.	z	P>|z|	[95% conf.	interval]
sexcode						
F	2.535333	1.204659	2.10	0.035	.174245	4.896421
M	-.6625413	.9833888	-0.67	0.500	-2.589948	1.264865

Table 3: Regression summary delta version (FPP)

Linear regression

Number of obs	=	33
Replications	=	100
Wald chi2(4)	=	10.09
Prob > chi2	=	0.0389
R-squared	=	0.1823
Adj R-squared	=	0.0655
Root MSE	=	4.2220

delta_ve~fpp	Observed coefficient	Bootstrap std. err.	z	P>|z|	Normal-based [95% conf.	interval]
ns_ver	.4058231	.1456059	2.79	0.005	.1204408	.6912054
bmi	.0838532	.1675331	0.50	0.617	-.2445056	.412212
ageatsurgery	.042833	.0876892	0.49	0.625	-.1290347	.2147007
sexcode						
M	-1.069394	1.513191	-0.71	0.480	-4.035193	1.896405
_cons	-11.15331	8.028162	-1.39	0.165	-26.88822	4.581602

Table 4: Regression summary delta total offset

Linear regression

Number of obs	=	33
Replications	=	100
Wald chi2(4)	=	8.13
Prob > chi2	=	0.0871
R-squared	=	0.3269
Adj R-squared	=	0.2308
Root MSE	=	2.5558

delta_tota~t	Observed coefficient	Bootstrap std. err.	z	P>|z|	Normal-based [95% conf.	interval]
ns_offset	.3873898	.152568	2.54	0.011	.0883621	.6864175
bmi	-.1056797	.0965304	-1.09	0.274	-.2948758	.0835164
ageatsurgery	.0361236	.0486822	0.74	0.458	-.0592917	.131539
sexcode						
M	1.436563	.9589039	1.50	0.134	-.4428545	3.31598
_cons	.9346356	3.761913	0.25	0.804	-6.438579	8.30785

Table 5: Regression summary delta leg length difference

```
Linear regression                                 Number of obs  =      33
                                                  Replications   =     100
                                                  Wald chi2(4)   =    3.95
                                                  Prob > chi2    =  0.4121
                                                  R-squared      =  0.1417
                                                  Adj R-squared  =  0.0190
                                                  Root MSE       =  2.7817
```

delta_tota~d	Observed coefficient	Bootstrap std. err.	z	P>|z|	Normal-based [95% conf.	interval]
ns_lld	.3422847	.2039923	1.68	0.093	-.0575329	.7421023
bmi	-.1579938	.1404499	-1.12	0.261	-.4332704	.1172829
ageatsurgery	-.0949432	.0825915	-1.15	0.250	-.2568195	.0669332
sexcode						
M	-.1577911	.9626075	-0.16	0.870	-2.044467	1.728885
_cons	9.186773	8.249123	1.11	0.265	-6.98121	25.35476
