# Supplementary material for: Imageless navigation system (Naviswiss) provides accurate component position in total hip arthroplasty with lateral decubitus position for end-stage hip osteoarthritis: a prospective cohort study with CT-validation
Source: Arthroplasty. 2024 Jan 8;6:3. doi: 10.1186/s42836-023-00224-0 (PMC10773062; doi:10.1186/s42836-023-00224-0)
Supplement: Supplementary file 3 — Additional file 3: Supplementary material 3. Summary of validation findings. [file 42836_2023_224_MOESM3_ESM.pdf]

Supplementary material 3 - Summary of validation findings

	This study	This study (absolute)	Hasegawa et al 2022[1] (supine - absolute)	Pooled[1-8] (N = 688)
Inclination_ FPP (°)	1.0 (4.6)	3.6 (3.1)		
Inclination (Bias Corrected)	0 (4.0)	3.2 (2.7)	2.8 (2.2)	2.8 (2.0)
Version_ FPP (°)	2.0 (4.5)	4.0 (2.6)		
Version (Bias Corrected)	0 (4.0)	3.4 (2.2)	2.8 (2.0)	3.6 (3.5)
Offset (mm)*	2.1 (2.4)	2.4 (2.1)		
LLD (mm)*	0.4 (2.4)	1.8 (1.3)		

*Declarations omitted

1. Hasegawa, M., Naito, Y., Tone, S. & Sudo, A. Accuracy of a novel accelerometer-based navigation (Naviswiss) for total hip arthroplasty in the supine position. *BMC Musculoskelet. Disord.* **23**, 537 (2022).
2. Tetsunaga, T. *et al.* Comparison of the accuracy of CT- and accelerometer-based navigation systems for cup orientation in total hip arthroplasty. *Hip Int.* **31**, 603–608 (2020).
3. Hasegawa, M., Tone, S., Naito, Y., Wakabayashi, H. & Sudo, A. Comparison of the accuracies of computed tomography-based navigation and image-free navigation for acetabular cup insertion in total hip arthroplasty in the lateral decubitus position. *Comput Assist Surg (Abingdon)* **26**, 69–76 (2021).
4. Iwana, D. *et al.* Accuracy of angle and position of the cup using computed tomography-based navigation systems in total hip arthroplasty. *Comput. Aided Surg.* **18**, 187–194 (2013).
5. Nakahara, I., Kyo, T., Kuroda, Y. & Miki, H. Effect of improved navigation performance on the accuracy of implant placement in total hip arthroplasty with a CT-based navigation system. *J. Artif. Organs* **21**, 340–347 (2018).
6. Ueoka, K. *et al.* The Accuracy of the Computed Tomography-Based Navigation System in Total Hip Arthroplasty Is Comparable With Crowe Type IV and Crowe Type I Dysplasia: A Case-Control Study. *J. Arthroplasty* **34**, 2686–2691 (2019).
7. Naito, Y., Hasegawa, M., Tone, S., Wakabayashi, H. & Sudo, A. The accuracy of acetabular cup placement in primary total hip arthroplasty using an image-free navigation system. *BMC Musculoskelet. Disord.* **22**, 1016 (2021).
8. Tsukamoto, M., Kawasaki, M., Suzuki, H., Fujitani, T. & Sakai, A. Proposal of accurate cup placement procedure during total hip arthroplasty based on pelvic tilt discrepancies in the lateral position. *Sci. Rep.* **11**, 13870 (2021).
